# Team‐based POCUS resuscitation simulation: Transformative learning for students

**DOI:** 10.1111/medu.15625

**Published:** 2025-02-19

**Authors:** Sin‐Yee Patty Kwong, Shiuan‐Ruey Yu, Yi‐Ting Chen, Chung‐Hsien Chaou

## 1 WHAT PROBLEM WAS ADDRESSED

The increasing complexity of modern medical practice presents challenges for medical students and novice physicians as they prepare for independent practice. Many recent graduates lack adequate practical skills and teamwork experience, particularly in high‐stakes areas like emergency resuscitation, where errors can result in serious consequences.^1^ Although physicians are expected to lead emergency teams, traditional medical training rarely emphasizes the critical teamwork required for effective resuscitation. Additionally, while Point‐of‐Care Ultrasound (POCUS) has become a vital tool in emergency medicine, structured, scenario‐based training is often lacking. This study addresses these gaps by exploring the effectiveness of POCUS‐integrated, team‐based simulation training in improving students' competency and confidence.

## 2 WHAT WAS TRIED?

The training programme, designed by a panel of Emergency Medicine and POCUS experts, consisted of three phases. First, students engaged in online multimedia flipped learning, accessing pre‐recorded ultrasound videos on topics related to POCUS and allowing them to study at their own pace. In the second phase, students participated in ultrasound training with standardized patients, focusing on mastering ultrasound techniques for diagnosing heart, lung, abdomen and vascular conditions. Finally, the third phase involved team‐based simulation scenarios, where students practiced team resuscitation in simulated emergencies, including myocardial infarction, cardiac tamponade, haemorrhagic shock and pneumothorax. All cases involved a cardiac arrest and resuscitation process. The course was facilitated by one senior emergency physician instructor and one standardized nurse. The learners were rated using standardized checklists during each simulation, and debriefing and feedback were provided right after each case. Quantitative and qualitative data were collected for programme evaluation.

## 3 WHAT LESSONS WERE LEARNED?

In contrast to traditional training, students found simulation‐based learning to be far more effective in capturing the complexity of real‐life medical emergencies. Quantitative data showed statistically significant improvements in students' self‐efficacy in handling emergency situations. Medical students also experienced a significant boost in their clinical skills and critical thinking abilities across all areas (p < 0.0001). Qualitative data showed that hands‐on simulation learning bridges the gap between theoretical knowledge and real‐world clinical application. It helped students develop a deeper understanding of the key elements required for effective emergency management, including teamwork, communication and situational awareness. Some mentioned that it transformed them from passive observers into active participants and decision‐makers. This outcome underscores the importance of simulating high‐pressure environments to prepare students for real‐world situations.

Many ultrasound learning sessions use standardized patients or high‐fidelity mannequins to create “hands‐on” practice opportunities, but they often lack the urgency and psychological pressure of real clinical situations. The inclusion of POCUS within resuscitation scenarios helps students recognize its diagnostic value, yet they are continually reminded that patient conditions can change at any moment. Besides integrating ultrasound data with other information obtained, timely and appropriate diagnoses and treatments must be given. This hands‐on experience with ultrasound reinforces the importance of using POCUS in emergency care. Students suggested that more simulation cases and additional practice opportunities would be beneficial for mastering the skill, aligning with the principles of deliberate practice. Their feedback will be used to further refine future iterations of the course.

## AUTHOR CONTRIBUTIONS

**Sin‐Yee Patty Kwong:** Writing – original draft; project administration; data curation; writing – review and editing. **Shiuan‐Ruey Yu:** Data curation; conceptualization; project administration; formal analysis; validation; funding acquisition. **Yi‐Ting Chen:** Data curation; project administration. **Chung‐Hsien Chaou:** Conceptualization; methodology; supervision; writing – review and editing; project administration; funding acquisition; data curation; formal analysis.

## CONFLICT OF INTEREST STATEMENT

The authors declare that they have no competing interests.

## ETHICS APPROVAL

The study was approved by the local ethical review board (IRB No. 202200134B0A3). Informed consent was obtained from all individual participants included in the study.

## Data Availability

The data that support the findings of this study are available from the corresponding author upon reasonable request.

## References

[1] Dyre L , Tabor A , Ringsted C , Tolsgaard MG . Imperfect practice makes perfect: error management training improves transfer of learning. Med Educ. 2017;51(2):196‐206. doi:10.1111/medu.13208 27943372

